# A genome-wide association study of total child psychiatric problems scores

**DOI:** 10.1371/journal.pone.0273116

**Published:** 2022-08-22

**Authors:** Alexander Neumann, Ilja M. Nolte, Irene Pappa, Tarunveer S. Ahluwalia, Erik Pettersson, Alina Rodriguez, Andrew Whitehouse, Catharina E. M. van Beijsterveldt, Beben Benyamin, Anke R. Hammerschlag, Quinta Helmer, Ville Karhunen, Eva Krapohl, Yi Lu, Peter J. van der Most, Teemu Palviainen, Beate St Pourcain, Ilkka Seppälä, Anna Suarez, Natalia Vilor-Tejedor, Carla M. T. Tiesler, Carol Wang, Amanda Wills, Ang Zhou, Silvia Alemany, Hans Bisgaard, Klaus Bønnelykke, Gareth E. Davies, Christian Hakulinen, Anjali K. Henders, Elina Hyppönen, Jakob Stokholm, Meike Bartels, Jouke-Jan Hottenga, Joachim Heinrich, John Hewitt, Liisa Keltikangas-Järvinen, Tellervo Korhonen, Jaakko Kaprio, Jari Lahti, Marius Lahti-Pulkkinen, Terho Lehtimäki, Christel M. Middeldorp, Jackob M. Najman, Craig Pennell, Chris Power, Albertine J. Oldehinkel, Robert Plomin, Katri Räikkönen, Olli T. Raitakari, Kaili Rimfeld, Lærke Sass, Harold Snieder, Marie Standl, Jordi Sunyer, Gail M. Williams, Marian J. Bakermans-Kranenburg, Dorret I. Boomsma, Marinus H. van IJzendoorn, Catharina A. Hartman, Henning Tiemeier

**Affiliations:** 1 Department of Child and Adolescent Psychiatry/Psychology, Erasmus University Medical Center, Rotterdam, The Netherlands; 2 Lady Davis Institute for Medical Research, Jewish General Hospital, Montreal, QC, Canada; 3 Department of Epidemiology, University of Groningen, University Medical Center Groningen, Groningen, The Netherlands; 4 COPSAC, Copenhagen Prospective Studies on Asthma in Childhood, Herlev and Gentofte Hospital, University of Copenhagen, Copenhagen, Denmark; 5 Steno Diabetes Center Copenhagen, Gentofte, Denmark; 6 Department of Medical Epidemiology and Biostatistics, Karolinska Institutet, Stockholm, Sweden; 7 Department of Epidemiology and Biostatistics, School of Public Health, Imperial College London, London, United Kingdom; 8 Telethon Kids Institute, University of Western Australia, Perth, Australia; 9 Netherlands Twin Register, Dept Biological Psychology, Vrije Universiteit, Amsterdam, The Netherlands; 10 Australian Centre for Precision Health, University of South Australia Cancer Research Institute, School of Health Sciences, University of South Australia, Adelaide, Australia; 11 South Australian Health and Medical Research Institute, Adelaide, Australia; 12 Child Health Research Centre, University of Queensland, Brisbane, QLD, Australia; 13 Biological Psychology, Vrije Universiteit Amsterdam, Amsterdam, The Netherlands; 14 Amsterdam Public Health Research Institute, Amsterdam, The Netherlands; 15 Centre for Life-Course Health Research, University of Oulu, Oulu, Finland; 16 Social, Genetic, and Developmental Psychiatry Centre, Institute of Psychiatry, Psychology and Neuroscience, King’s College London, London, United Kingdom; 17 Institute for Molecular Medicine Finland (FIMM), University of Helsinki, Helsinki, Finland; 18 MRC Integrative Epidemiology Unit, Department of Population Health Sciences, Bristol Medical School, University of Bristol, Bristol, United Kingdom; 19 Language and Genetics Department, Max Planck Institute for Psycholinguistics, Nijmegen, The Netherland; 20 Donders Institute for Brain, Cognition and Behaviour, Radboud University, Nijmegen, The Netherlands; 21 Department of Clinical Chemistry, Fimlab Laboratories, Tampere, Finland; 22 Department of Clinical Chemistry, Finnish Cardiovascular Research Center—Tampere, Faculty of Medicine and Health Technology, Tampere University, Tampere, Finland; 23 Department of Psychology and Logopedics, Faculty of Medicine, University of Helsinki, Helsinki, Finland; 24 Center for Genomic Regulation (CRG), The Barcelona Institute of Science and Technology, Barcelona, Spain; 25 BarcelonaBeta Brain Research Center (BBRC)–Pasqual Maragall Foundation, Barcelona, Spain; 26 Department of Clinical Genetics, Erasmus Medical Center, Rotterdam, The Netherlands; 27 Universitat Pompeu Fabra (UPF), Barcelona, Spain; 28 Institute of Epidemiology, Helmholtz Zentrum München—German Research Center for Environmental Health, Neuherberg, Germany; 29 LMU–Ludwig-Maximilians-Universität Munich, Div. Metabolic and Nutritional Medicine, Dr. von Hauner Children’s Hospital, University of Munich Medical Center, Munich, Germany; 30 School of Medicine and Public Health, Faculty of Medicine and Health, The University of Newcastle, Newcastle, New South Wales, Australia; 31 Division of Substance Dependence, Department of Psychiatry, University of Colorado Anschutz Medical Campus, Aurora, CO, United States of America; 32 Institute for Behavioral Genetics, University of Colorado Boulder, Boulder, CO, United States of America; 33 ISGlobal, Centre for Research in Environmental Epidemiology (CREAL), Barcelona, Spain; 34 CIBER Epidemiology and Public Health (CIBERESP), Barcelona, Spain; 35 Avera Institute for Human Genetics, Sioux Falls, South Dakota, United States of America; 36 Institute of Molecular Bioscience, University of Queensland, Brisbane, QLD, Australia; 37 Population, Policy and Practice, UCL Great Ormond Street Institute of Child Health, London, United Kingdom; 38 Department of Pediatrics, Naestved Hospital, Naestved, Denmark; 39 Institute and Clinic for Occupational, Social and Environmental Medicine, University Hospital, LMU Munich, Munich, Germany; 40 Department of Psychology and Neuroscience, University of Colorado Boulder, Boulder, CO, United States of America; 41 Department of Public Health, University of Helsinki, Helsinki, Finland; 42 Child and Youth Mental Health Service, Children’s Health Queensland Hospital and Health Service, Brisbane, QLD, Australia; 43 Public Health, University of Queensland, Brisbane, QLD, Australia; 44 Interdisciplinary Center Psychopathology and Emotion Regulation (IPCE), University of Groningen, University Medical Center Groningen, Groningen, The Netherlands; 45 Department of Clinical Physiology and Nuclear Medicine, Turku University Hospital, Turku, Finland; 46 Research Centre of Applied and Preventive Cardiovascular Medicine, University of Turku, Turku, Finland; 47 IMIM (Hospital del Mar Medical Research Institute), Barcelona, Spain; 48 Clinical Child & Family Studies, Vrije Universiteit Amsterdam, The Netherlands; 49 School of Clinical Medicine, University of Cambridge, Cambridge, United Kingdom; 50 Department of Psychiatry, University of Groningen, University Medical Center Groningen, Groningen, The Netherlands; 51 Department of Social and Behavioral Science, Harvard TH Chan School of Public Health, Boston, MA, United States of America; University of Oxford, UNITED KINGDOM

## Abstract

Substantial genetic correlations have been reported across psychiatric disorders and numerous cross-disorder genetic variants have been detected. To identify the genetic variants underlying general psychopathology in childhood, we performed a genome-wide association study using a total psychiatric problem score. We analyzed 6,844,199 common SNPs in 38,418 school-aged children from 20 population-based cohorts participating in the EAGLE consortium. The SNP heritability of total psychiatric problems was 5.4% (SE = 0.01) and two loci reached genome-wide significance: rs10767094 and rs202005905. We also observed an association of *SBF2*, a gene associated with neuroticism in previous GWAS, with total psychiatric problems. The genetic effects underlying the total score were shared with common psychiatric disorders only (attention-deficit/hyperactivity disorder, anxiety, depression, insomnia) (rG > 0.49), but not with autism or the less common adult disorders (schizophrenia, bipolar disorder, or eating disorders) (rG < 0.01). Importantly, the total psychiatric problem score also showed at least a moderate genetic correlation with intelligence, educational attainment, wellbeing, smoking, and body fat (rG > 0.29). The results suggest that many common genetic variants are associated with childhood psychiatric symptoms and related phenotypes in general instead of with specific symptoms. Further research is needed to establish causality and pleiotropic mechanisms between related traits.

## Introduction

Psychiatric disorders are moderately heritable, on average about 30–50% of the variability in symptoms can be explained by genetic differences between individuals [1]. The joint effect of common single nucleotide polymorphisms (SNP heritability) explains 5% to 30% of the variance in psychiatric disorders in adults [2]. Similar levels have been reported for behavioral and emotional symptoms in children, although there is large variability depending on child age and informant [3, 4]. A focus on childhood problems is particularly important, as many adult disorders can be traced back to problems in childhood [5].

Recent family and molecular genetic studies demonstrated that much of the genetic effects underlying psychiatric disorders are not unique to particular diagnoses, but rather shared across several psychiatric diagnoses and symptoms [2, 6–10]. This phenomenon is known as cross-phenotype association and suggests pleiotropy, i.e. the influence of a genetic variant on multiple traits, [11] and may be an explanation for the extensive co-occurrence of mental disorders [12]. Several lines of evidence support this notion. First, the SNP based genetic correlations between disorders from different domains, such as major depression, attention-deficit/hyperactivity disorder (ADHD), bipolar disorder and schizophrenia are moderate to high, [2] averaging 0.41 [10]. Second, measures of global psychopathology in children showed a common SNP heritability between 16% and 38% [8, 13]. Third, a genome-wide association meta-analysis (GWAS) of eight psychiatric disorders (ADHD, anorexia, autism, bipolar, depression, obsessive compulsive disorder, schizophrenia and Tourette’s) identified 23 loci associated with at least four of these disorders [14].

GWAS derived polygenic risk scores (PRS) for single disorders are good predictors of general psychopathology. For instance, a PRS for ADHD was more strongly associated with a general psychopathology factor than with specific hyperactivity or attention problems adjusted for general psychopathology [15]. In another study a composite PRS based on eight GWAS was associated with general psychopathology in childhood [16]. These cross-phenotype associations present a challenge in interpreting GWAS results that typically target a single disorder, raising the question of whether a multi-disorder approach would be more informative.

Previous GWAS of childhood disorders, such as autism spectrum disorders, ADHD, aggression and internalizing disorders, [4, 17–19] have provided insights into the genetic architecture of child psychiatric problems and into the genetic correlations between childhood psychiatric problems. However, with notable exceptions of a large recent ADHD study [20] and a GWAS on autism spectrum disorder, [17] these studies mostly failed to identify individual genome-wide significant loci. Besides increasing the sample size, some researchers propose the inclusion of related phenotypes in analyses to increase power [21, 22]. Genetic loci with pleiotropic effects may be missed in a GWAS of single psychiatric disorders. If a variant only modestly increases the risk of symptoms from different domains, any association with a specific disorder may be too weak to be detected. A focus on global psychopathology increases the power to detect unspecific genetic loci, which are associated with global psychiatric vulnerabilities. A previous GWAS [14] examined multiple disorder simultaneously, but analyses of multiple dimensional measures of psychiatric problems in childhood are lacking. This approach is arguably particularly promising in childhood given the less clearly expressed symptoms and the low homotypic but high heterotypic stability of problems, [23] i.e. the changing of symptoms from one domain to another.

Our aim was to identify genetic loci associated with a total psychiatric problem score representing a variety of psychiatric problems including internalizing, externalizing, attention, neurodevelopmental and other psychiatric problems. To identify these genetic variants, we performed a GWAS meta-analysis within the EArly Genetics and Lifecourse Epidemiology (EAGLE) consortium (https://www.eagle-consortium.org/). Finally, we estimated genetic correlations of the total psychiatric problem score with various single child and adult psychiatric, psychological, neurological and lifestyle or educational characteristics.

## Methods and materials

### Participants

Cohorts from the EAGLE consortium with parent-rated measures of psychiatric symptoms in the age range 5–16 years were invited to participate in the project Twenty cohorts from Europe, the US and Australia contributed data to this meta-analysis. See Table 1 and S1 File for cohort descriptions. Parents provided written informed consent for their children’s participation and the study was approved by the Ethics Committee of Erasmus MC, as well as by local ethics committees at each site, see S1 File for full ethics statements per participating cohort. The study was performed in accordance with the Declaration of Helsinki. We restricted the analysis to children of European ancestry to avoid population stratification bias. In total data from 38,418 participants with a mean age of 9.9 years (SD = 2.02) were meta-analyzed. This study was originally planned with a discovery-replication design. However, the obtained sample-size was not sufficiently large to split the sample, and we opted for maximizing power in discovery analyses.

**Table 1:** Phenotype characteristics.

Cohort	n	Instrument	Domains	Informant	Age years	Age SD	Score Mean	Score SD	% Female
1958BC-T1DGC	2170	Rutter	Int,Ext	Maternal	11.3	0.1	6.2	3.4	51
1958BC-WTCCC	2261	Rutter	Int,Ext	Maternal	11.3	0.2	6.2	3.4	48
ALSPAC	5461	SDQ	Int,Ext	Maternal	9.6	0.1	6.7	4.8	49
BREATHE	1618	SDQ	Int,Ext	Both	8.3	3.9	8.1	5.1	48
CADD	358	CBCL 4–18	Int,Ext,Sleep,TP,EP,PDD	Both	13.0	2.6	16.2	21.9	28
CATSS	6498	A-TAC	Int,Ext,EP,PDD	Both	12.0	0.0	5.4	7.5	49
COPSAC2010	547	SDQ 4–10	Int,Ext	Both	6.0	0.3	7.1	4.7	48
FinnTwin12	959	MPNI	Int,Ext	Both	11.4	0.3	11.3	6.8	53
GenR	1847	CBCL 6–18	Int,Ext,Sleep,TP,EP,PDD	Maternal	9.7	0.3	17.3	15.2	51
Gini-Lisa	1389	SDQ	Int,Ext	Maternal	10.0	0.2	7.3	5.2	48
Glaku	312	CBCL 6–18	Int,Ext,Sleep,TP,EP,PDD	Maternal	12.1	1.0	21.7	16.8	52
INMA	745	SDQ	Int,Ext	Both	5.1	0.8	8.9	5.0	38
MUSP	1156	CBCL 6–18	Int,Ext,Sleep,TP,EP,PDD	Maternal	13.9	0.3	30.5	19.8	61
NFBC1986	3346	Rutter	Int,Ext	Maternal	7.8	0.2	2.6	2.1	51
NTR I	2563	CBCL 6–18	Int,Ext,Sleep,TP,EP,PDD	Maternal	9.9	1.0	19.3	15.9	52
NTR II	2960	CBCL 6–18	Int,Ext,Sleep,TP,EP,PDD	Maternal	9.6	1.0	19.1	16.6	53
RAINE	1366	CBCL 4–18	Int,Ext,Sleep,TP,EP,PDD	Both	10.6	0.2	21.1	18.6	48
TCHAD	2111	CBCL 6–18	Int,Ext,Sleep,TP,EP,PDD	Both	13.0	0.0	11.7	12.5	51
TEDS	2707	SDQ	Int,Ext	Both	11.3	0.7	7.0	5.0	54
TRAILS	1283	CBCL 6–18	Int,Ext,Sleep,TP,EP,PDD	Maternal	11.1	0.6	0.2	0.2	52
YFS	1352	HES	Int,Ext	Maternal	10.6	3.3	14.7	6.8	54

**n** sample size

**Domains** covered by instrument: Internalizing (Int), Externalizing (Ext), Sleep, Thought Problems (TP), Eating Problems (EP), Pervasive Developmental Disorder Score (PDD)

**Informant** questionnaire filled in by only mothers (maternal) or by either father or mother (both)

**SD** standard deviation

### Outcome

Psychiatric problems were assessed with parent-rated questionnaires at the assessment wave closest to age 10 years. All items of a broad psychiatric questionnaire were summed into a single total psychiatric sum score. In all cohorts internalizing, externalizing and attention problems were assessed; in some questionnaires items on sleep, thought, eating problems, and pervasive developmental disorders were included in the total problem score (Table 1). Instruments included the Child Behavior Checklist (CBCL), [24] Strengths and Difficulties Questionnaire (SDQ), [25] parental version of the Multidimensional Peer Nomination Inventory (MPNI), [26] Rutter Children’ Behaviour Questionnaire, [27] the Autism–Tics, AD/HD and other Comorbidities inventory (A-TAC) [28], and items derived from the Health Examination Survey [29].

We applied a log transformation plus 1 to avoid bias due to non-normal residuals and influential observations. Because different scales were used, the log-transformed scores were converted to a z-score within cohorts to make units comparable across cohorts.

### Genotyping and QC

Genotyping was performed using genome-wide arrays. Cohort-specific pre-imputation quality control (QC) was performed using established protocols. In all cohorts, SNPs were imputed to the 1000 Genomes Phase 1 or Phase 3 reference panel [30]. Each cohort performed a GWAS and summary results were collected for meta-analysis. We omitted the X-chromosome from further analysis as most cohorts had no information available on X-linked SNPs. Pre-meta-analysis QC was performed with EasyQC and QCGWAS [31–33]. The QC steps are summarized in S1 Fig. After meta-analysis, we excluded SNPs with low minor allele frequency (MAF < 5%), sample size (<5000), or with data from a small number of cohorts (<5). Finally, we checked the pooled results for spurious inflation by examining QQ-plots of the p-value distribution and by examining the LD score regression intercept (see statistical analysis). Full genetic methods and quality control per cohort can be found in S1 and S2 Tables.

### Statistical analysis

#### Single SNP associations and meta-analysis

The z-scores of the total psychiatric problems scores were related to the SNP dosages in a linear model. Covariates included gender, age at assessment and principal components of ancestry. The number of dimensions (1–10) were specified by each cohort. CATSS and TCHAD additionally used a random effect to account for familial relatedness. FinnTwin12 and NTR applied a mixed model with two random effects to control for population stratification and relatedness. We pooled the results from the individual cohorts using an inverse-variance weighted fixed-effects meta-analysis. R 3.4.3 was used for QC, data preparation and analysis of results [34]. Meta-soft 2.0.1 was used for the meta-analysis of single SNP associations [35]. The individual cohort results after quality control were examined and meta-analyzed independently by the first and second author with consistent results. Genome-wide significance was set at p<5E-08. We had good power (>80%) to detect effect sizes between 0.05 for MAF = 50% and 0.11 for MAF = 5%. Effect size (Beta) was defined as change in log(total problems+1) in SD per effect allele. Power was calculated with gwas-power (https://github.com/kaustubhad/gwas-power) [36].

We also used the FUMA web tool [37] to explore potential functional implications of any identified variants. We reviewed positional mapping, eQTL analyses and chromatin interactions with all available databases (date: 2019-06-30). We also performed a lookup in the mQTL [38] database, to check for potential influences on gene expression via DNA methylation.

#### Gene-based and expression analysis

We performed gene-based tests using MAGMA [39] in FUMA. MAGMA estimates the joint effect of all SNPs within a gene, while accounting for the LD structure and gene size. We tested 18,168 protein coding genes and thus the p-value significance threshold was set at 3e-6 based on Bonferroni correction.

Second, we tested, whether the results from the gene-based tests were related to gene expression in several tissues. Specifically, we used MAGMA to test whether the strength of association between genes and the total psychiatric problem score was related to the mean gene expression level in a specific tissue, while considering average expression levels. Given that we expected gene variants to act via brain pathways, we tested expression in 13 brain regions (S3 Table). However, as gene effects may impact the brain indirectly via other tissues, we also investigated gene expression levels on an organ level (S4 Table). Gene expression levels were obtained from the GTEx 7 database [40].

Third, we further examined whether the predicted gene expression of selected genes was related to total psychiatric problems. We selected genes, that were (functionally) annotated to genome-wide hits, or that were genome-wide significant according to gene-based tests. To correlate gene expression with total psychiatric problems, we used a transciptome-wide association study (TWAS) approach [41]. In short, gene expression in a tissue is imputed based on expression information from the GTEx 7 database for a specific tissue and then correlated with a phenotype, as inferred from GWAS summary statics. We chose to examine the expression in the basal ganglia post-hoc, as the loci most strongly associated with total psychiatric problems were related to genes expressed in the basal ganglia regions, see section “results: gene expression” below. We also performed a lookup on TWAS hub, to examine whether gene expression by a gene identified in this study has previously been associated with other phenotypes [42, 43].

#### SNP heritability and genetic correlations

We estimated the SNP heritability of total psychiatric problem scores with LD score regression [44]. We used the online tool LD Hub [45] to estimate common SNP heritability and genetic correlations with various psychiatric, psychological, neurological and lifestyle or educational characteristics. To compute the genetic correlations we selected published GWAS summary statistics available on LD Hub, except genetic correlations with anxiety symptoms, [46] which were computed locally with ldsc 1.0.0.

#### Sensitivity analyses

The GWAS meta-analysis included cohorts using different instruments to measure total psychiatric problems with mean age ranges from 5 to 13 years. While our inclusion criteria maximized sample sizes and also increased the potential for generalizability, this approach may have increased the study heterogeneity. We therefore performed two sensitivity analyses to investigate effect consistency. In the first, we performed three separate meta-analyses for each of the major instrument groups. These meta-analyses included only cohorts using the CBCL, SDQ or Rutter questionnaires, respectively. We then tested consistency of genetic effects by computing the genetic correlation between each instrument.

In the second sensitivity analyses, we tested, whether the genetic correlation between total psychiatric problems and thought disorders changed depending on the age at which total psychiatric problems were assessed. Specifically, we hypothesized, that total psychiatric problems scores assessed during early adolescence would correlate more strongly with schizophrenia and bipolar disorders, than total psychiatric problems occurring before the onset of puberty. We therefore performed two meta-analyses consisting of the cohorts assessing youths below and equal or above age 12 years. We then examined the genetic correlation of total psychiatric problems in both age groups with schizophrenia and bipolar disorder.

## Results

### Spurious inflation and SNP Heritability

**Fig 1:**
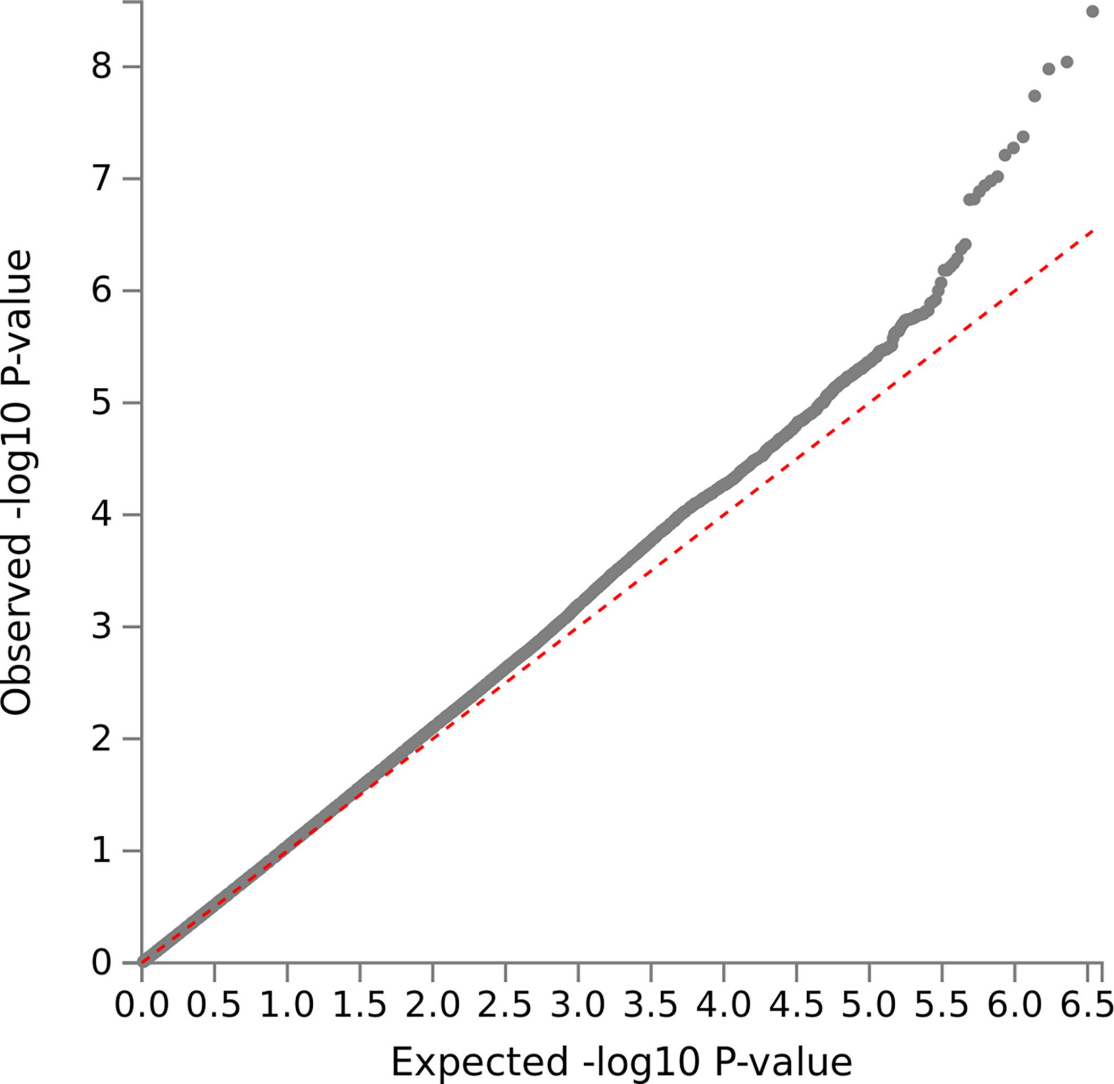
QQ-plot. Quantile-quantile plot of observed -log 10 p values vs expected -log 10 p values assuming chance findings in single SNP analysis. Diagonal line indicates a p value distribution compatible with chance finding. Upward deviations indicate p values more significant than expected.

We tested 6,844,199 SNPs after quality control. The QQ-plot (Fig 1) showed some inflation, however, the LD score intercepts was close to 1 (β_0_ = 1.01, SE = 0.01), suggesting that the inflation was due to a true signal rather than spurious associations. The SNP heritability was estimated at 5.4% (SE = 0.013).

#### SNP based tests

**Fig 2:**
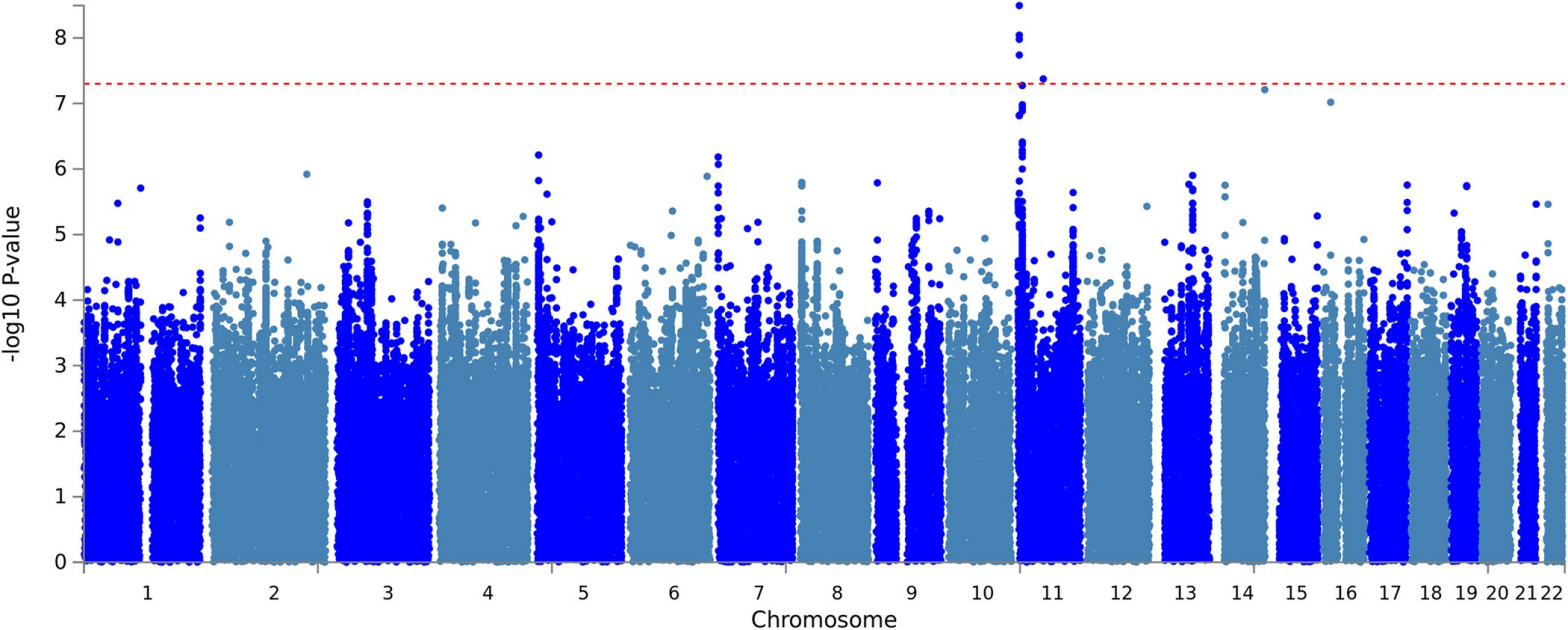
Manhattan plot. Manhattan plot of -log 10 p values vs SNP position for single SNP analysis. SNPs above the red horizontal line indicate genome-wide significant findings.

Two loci on chromosome 11 were genome-wide significant, see Fig 2. One locus is located around lead SNP rs10767094, which showed an increase of 0.08SD in total psychiatric problems per A allele (SE = 0.01, p = 3E-09, n = 8,216) (S2 Fig). The A allele is very common with an average frequency of 48% across the cohorts, but the SNP’s average imputation quality was a moderate 50% (Info/R^2^). Information on this locus was only available in 27% of participants (6 cohorts). The SNP showed a moderate amount of effect heterogeneity (I^2^ = 47.6%). Also on chromosome 11 an insertion/deletion variant (InDel) was genome-wide significant. A deletion of the A allele at rs202005905 was associated with an increase of 0.08SD in total psychiatric problems (SE = 0.01, p = 4E-08, n = 15,886, S3 Fig). Deletion prevalence was on average 16%, but again the imputation quality was modest with 52%, information was available in 41% of participants (9 cohorts) and the genetic variant showed moderate effect heterogeneity (I^2^ = 59.6%).

The SNP rs10767094 lies in the intron of the long non-coding RNA Loc105379880 and rs202005905 lies in an intergenic region with no nearby genes. A FUMA eQTL and chromatin interaction analysis did not reveal any interactions with genes. The mQTL database did not list any associations with DNA methylation.

The third top locus did not reach genome-wide significance, but is of interest for its location in a gene previously implicated in neuroticism [47, 48] as well as being very close to genome-wide significance. The SNP rs72854494 lies within the gene *SBF2*. The T allele was associated with 0.05SD lower total psychiatric problems (SE = 0.01, p = 5E-08, n = 38,330) (S4 Fig). This association showed no heterogeneity (I^2^ = 0.0%) among the cohorts. The T allele occurred on average in 14% across cohorts, with a very good imputation quality of 96%. FUMA eQTL and chromatin interaction analysis, as well as a lookup in mQTL DB did not reveal any further information on functional association. Results for all SNPs with genome-wide suggestive p-values (p<5E-06) can be found in Table 2. The full summary statistics can be found at https://doi.org/10.6084/m9.figshare.17170994.v1.

**Table 2:** SNPs with genome-wide significant (p<5E-08) and suggestive (p<5E-07) results.

SNP	Chr	BP	EA	OA	EAF	n_stu_	n	β	SE	p	I^2^
rs10767094	11	3477509	A	G	0.48	6	8216	0.08	0.01	3E-09	47.6
rs12098951	11	3478953	A	G	0.48	8	10417	0.08	0.01	9E-09	52.3
rs10767093	11	3477421	T	A	0.48	8	10408	0.08	0.01	1E-08	40.3
rs10767096	11	3477891	T	C	0.53	8	10382	0.07	0.01	2E-08	47.6
rs202005905	11	54733705	I	D	0.84	9	15886	-0.08	0.01	4E-08	59.6
rs72854494	11	9946312	T	C	0.86	21	38330	-0.05	0.01	5E-08	0.0
rs188216744	14	106478354	T	C	0.55	5	7045	0.08	0.01	6E-08	87.2
rs115749482	16	16754648	A	G	0.81	5	6930	0.08	0.01	1E-07	85.6
rs59076561	11	9951438	G	T	0.86	20	35645	-0.05	0.01	1E-07	0.0
rs113227893	11	9944120	D	I	0.86	18	33850	-0.05	0.01	1E-07	0.2
rs67456791	11	9944108	G	A	0.86	20	35533	-0.05	0.01	1E-07	0.0
rs10767095	11	3477568	G	A	0.48	8	10413	0.07	0.01	2E-07	43.8
rs10834158	11	3477887	A	G	0.52	9	11682	0.07	0.01	2E-07	56.1
rs116657155	11	9954242	A	G	0.85	20	35619	-0.05	0.01	4E-07	0.0
rs60713856	11	9955418	G	C	0.85	20	35579	-0.05	0.01	4E-07	0.0
rs140557414	11	9953387	A	C	0.85	20	35632	-0.05	0.01	5E-07	0.0
rs57331333	11	9956272	T	C	0.85	20	35570	-0.05	0.01	6E-07	0.0
rs34543113	5	3339568	G	A	0.70	20	35612	-0.04	0.01	6E-07	0.0
rs11042555	11	9957159	T	C	0.85	20	35571	-0.05	0.01	7E-07	0.0
rs36189439	7	323206	A	G	0.58	6	7814	0.07	0.01	7E-07	69.2

**Chr** Chromosome

**BP** Basepair Position (Build 37 map)

**EA** Effect Allele

**OA** Other Allele

**EAF** Effect Allele Frequency

**n**_**stu**_ Number of Studies

**n** Sample Size

**β** Beta

**SE** standard error

**p** p-value

**I**^**2**^ Effect heterogeneity

### Gene-based test

Next we tested the association of 18,290 protein coding genes with the child total psychiatric problem score. None of the genes reached genome-wide significance (S3 Table, S5 and S6 Figs). We also post-hoc looked up the association of *SBF2*. The aggregate of 1,508 SNPs in *SBF2* showed a nominal significance of p = 0.0004 (n = 35,736).

### Gene expression

We performed a MAGMA tissue expression analysis in 13 specific brain tissues (S4 Table). Genes more strongly associated with total psychiatric problems tended to express particularly in four subcortical structures: caudate, putamen, anterior cingulate cortex and amygdala. However, these associations were not significant after correction for multiple testing. In addition, we analyzed tissue expression for 30 tissues on an organ level, see S5 Table. None of the organs had statistically significant associations, however, expression in the brain showed the strongest association (p = 0.06).

The top two genome-wide significant loci were not linked to a characterized gene, thus we decided to perform a TWAS analysis only for *SBF2*. We found that higher predicted levels of *SBF2* in the basal ganglia were related to higher scores of total psychiatric symptoms (Z = +2.33, p = 0.02) based on the best linear unbiased predictions (BLUP) of a random variable representing 489 SNPs. A lookup in the TWAS Hub database revealed, that predicted levels of *SBF2* gene products associate most with following phenotypes: neuroticism, body fat measures, red blood cell count, nervous feelings and worrying (http://twas-hub.org/genes/SBF2/).

#### Genetic correlation

Next we quantified the extent to which the genetic associations of child psychiatric problems scores were shared with other phenotypes. After adjustment for false discovery rate, insomnia, depressive symptoms, neuroticism, cigarettes smoked per day, body fat, body mass index, number of children, and age of smoking initiation all showed positive genetic correlations between 0.29 and 0.60 with the total psychiatric problem score (Table 3) based on the results of independent GWAS in adults. The highest correlation of total psychiatric problems was with ADHD, but this association did not survive multiple testing correction (r_G_ = 0.86, SE = 0.39, p = 0.03, q = 0.06). Subjective wellbeing, childhood IQ, college completion, years of schooling, intelligence and age of smoking initiation showed significant negative correlations with the total psychiatric problem score, ranging from -0.66 to -0.42. Of the psychiatric phenotypes tested, the less common psychiatric disorders like schizophrenia, bipolar disorder, autism spectrum disorder, and anorexia were not genetically correlated with the total psychiatric problem score (r_G_ < 0.01).

**Table 3:** Genetic correlations based on LD score regression.

Correlated trait	PMID	r_G_	SE	p	q	h^2^
*Psychiatry*						
ADHD	20732625	0.86	0.39	3E-02	6E-02	0.19
**Depressive symptoms**	**27089181**	**0.60**	**0.13**	**1E-06**	**9E-06**	**0.05**
Anxiety symptoms	26754954	0.60	0.26	3E-01	4E-01	0.26
**Insomnia**	**28604731**	**0.49**	**0.15**	**9E-04**	**3E-03**	**0.05**
Major depressive disorder	22472876	0.22	0.17	2E-01	3E-01	0.14
PGC cross-disorder analysis	23453885	0.07	0.11	5E-01	6E-01	0.16
Autism spectrum disorder	28540026	0.01	0.15	9E-01	1E+00	0.37
Schizophrenia	25056061	-0.03	0.07	7E-01	8E-01	0.45
Bipolar disorder	21926972	-0.16	0.11	1E-01	2E-01	0.43
Anorexia Nervosa	24514567	-0.17	0.12	1E-01	2E-01	0.31
*Neurology*						
Amyotrophic lateral sclerosis	27455348	0.30	0.23	2E-01	3E-01	0.04
Parkinsons disease	19915575	0.14	0.12	2E-01	3E-01	0.37
Alzheimers disease	24162737	-0.10	0.17	6E-01	6E-01	0.05
*Personality and Wellbeing*						
**Neuroticism**	**27089181**	**0.41**	**0.09**	**1E-05**	**8E-05**	**0.09**
Neo-conscientiousness	21173776	0.05	0.23	8E-01	9E-01	0.07
Neo-openness to experience	21173776	0.01	0.18	1E+00	1E+00	0.11
**Subjective well being**	**27089181**	**-0.46**	**0.12**	**1E-04**	**4E-04**	**0.02**
*Intelligence and educational attainment*						
Childhood IQ	23358156	-0.42	0.16	8E-03	2E-02	0.27
**Years of schooling**	**25201988**	**-0.56**	**0.11**	**3E-07**	**2E-06**	**0.11**
**Intelligence**	**28530673**	**-0.63**	**0.11**	**1E-08**	**2E-07**	**0.20**
**College completion**	**23722424**	**-0.66**	**0.11**	**3E-09**	**8E-08**	**0.08**
*Brain volume*						
Mean Hippocampus	25607358	0.01	0.18	1E+00	1E+00	0.15
Mean Thalamus	25607358	-0.06	0.20	8E-01	8E-01	0.11
Infant head circumference	22504419	-0.13	0.18	5E-01	6E-01	0.22
Intracranial Volume	25607358	-0.15	0.20	4E-01	6E-01	0.17
Mean Pallidum	25607358	-0.17	0.17	3E-01	4E-01	0.17
Mean Caudate	25607358	-0.18	0.14	2E-01	3E-01	0.25
Mean Accumbens	25607358	-0.24	0.25	4E-01	5E-01	0.09
Mean Putamen	25607358	-0.25	0.13	6E-02	1E-01	0.29
*General health behaviors/outcomes*						
**Cigarettes smoked per day**	**20418890**	**0.58**	**0.22**	**9E-03**	**2E-02**	**0.05**
**Body fat**	**26833246**	**0.48**	**0.12**	**5E-05**	**2E-04**	**0.11**
**Body mass index**	**20935630**	**0.30**	**0.09**	**1E-03**	**3E-03**	**0.19**
Sleep duration	27494321	-0.18	0.11	9E-02	2E-01	0.05
Age of smoking initiation	20418890	-0.64	0.25	1E-02	2E-02	0.05
*Parent’s age at death*						
Parent’s age at death	27015805	-0.20	0.16	2E-01	3E-01	0.03
*Reproduction*						
**Number of children ever born**	**27798627**	**0.30**	**0.11**	**5E-03**	**2E-02**	**0.02**

**Bold** rows indicate correlates with statistical significance after multiple testing correction

**PMID** PubMed ID, **r**_**G**_ Genetic Correlation, **SE** Standard Error, **p** P-value

**q** False Discovery Rate Adjusted P-values, **h**^**2**^ SNP heritability

### Sensitivity analyses

Total psychiatric problems measured with CBCL and SDQ genetically correlated with r_G_ = 0.84 (SE = 0.31, p = 0.008). The Rutter questionnaire correlated more modestly with both CBCL (r_G_ = 0.43, SE = 0.39, p = 0.073) and SDQ (r_G_ = 0.63, SE = 0.29, p = 0.030). The genetic correlations of total psychiatric problems assessed both before or after age 12 with schizophrenia and bipolar disorder were small and consistent with a null effect irrespective of age group (S6 Table).

## Discussion

The current study reports the first GWAS examining global psychopathology in children. Two genetic loci were genome-wide significant in the total sample. Additionally, we found support for the involvement of gene *SBF2* in the development of psychopathology. The genetic effects underlying global psychopathology were shared with common psychiatric disorders (ADHD, anxiety, depression, insomnia), but not with less common and on average more severe ones (schizophrenia, bipolar disorder, autism, eating disorders).

The two genome-wide significant variants are one SNP (rs10767094) and one InDel (rs202005905). To the best of our knowledge these variants have not been associated with psychiatric traits before. It is unclear, how exactly these variants or tagged causal variants may affect general psychopathology, as functional annotation for these loci is sparse. The modest imputation quality possibly affected study results as both variants failed quality control in most cohorts. Measurement error of the genotypes could explain the relatively high estimates heterogeneity. An important next step would therefore be to replicate these SNPs using direct genotyping or denser arrays.

While just not genome-wide significant, the evidence for an involvement of *SBF2* with the lead SNP rs72854494 in total psychiatric problems is more convincing. This locus has been implicated in neuroticism based on two GWAS. In a GWAS of neuroticism [47] rs1557341, located in *SBF2*, showed genome-wide significance. In a second larger independent GWAS of 449,484 participants, *SBF2* showed a genome-wide significant effect for both neuroticism and worry in gene-based tests [48]. Furthermore, according to TWAS hub, the predicted gene products of *SBF2* correlate with neuroticism based on several GWAS. Neuroticism describes a disposition to experience negative emotions and a higher stress reactivity. It robustly and substantially associates with general psychopathology in children, [8, 49] adolescence, [50] and adults [51] (between r = 0.13 and r = 0.81). A twin study suggested that this correlation arises partly due to shared genetic causes [52] and in this GWAS the genetic correlations between total psychiatric problems and neuroticism were substantial as well, similar to the phenotypic association (r_G_ = 0.41). These results suggest that *SBF2* pleiotropically affects neuroticism and psychopathology, but the mechanisms would need to be explored further. Neuroticism has been hypothesized to contribute strongly to general psychopathology, [53] thus it may mediate the effect of genetic variants on total psychiatric problems, but both phenotypes may also be independently affected. In regards to biology, human and mice studies points towards abnormal myelination as one of the consequences of *SBF2* alterations [54, 55]. We recently reported an association between lower global white matter integrity and higher levels of general psychopathology in school-aged children [56]. Thus, one may speculate that *SBF2* affects psychiatric problems via white matter development.

We additionally tested, whether genetic variants associated with total psychiatric problems were associated with gene expression in the brain. Association with gene expression in the limbic system of the brain showed the most support, but did not survive multiple testing correction. The findings are thus compatible with the possibility of a chance finding, but strong theoretical support for a major role of the limbic system exists. The limbic system includes evolutionary preserved regions responsible for emotion regulation and motivation, [57] which were previously implicated in affective disorders, ADHD and OCD, [58, 59] and are a potential intervention target [60].

In this study we observed 5% SNP heritability, which is similar to the LD score estimated SNP heritability of continuously measured ADHD, [4] depression, [47] and anxiety symptoms [46] in population based cohorts. The total psychiatric problem scores were based on various instruments, which all included items for common psychiatric internalizing, attention, and externalizing symptoms. Therefore, it is not surprising that common psychiatric symptoms and disorders such as ADHD and depression shared 36% or more of the genetic variation with the total psychiatric problem score. The extent to which the questionnaires used in this study covered other less common problems, such as psychotic, bipolar or autistic symptoms varied greatly by instrument. This may explain the low genetic correlation between total psychiatric problem scores with these disorders. Also, these disorders are less prevalent in the general population and thus may be reflected less in total problem scores. Furthermore, age of onset for schizophrenia and bipolar disorder is typically in late adolescence and early adulthood [61–63]. We did not find support for the notion that total psychiatric problems assessed in at later ages would genetically correlate more strongly with thought disorders than those assessed at younger ages. For autism spectrum disorder, the age of onset is early, but the prevalence in the cohorts was low. Thus, the total psychiatric problem score covered broad symptomatology but was not representative of severe psychiatric disorders with lower prevalence rates or emergence at later ages. The differential genetic correlations with common and relatively rare disorders suggests a continuum of genetic effects varying from very specific variants, variants which underlie either common or less common disorders, to variants which underlie most psychiatric problems. The presence of these universal variants is supported by genetic correlations between common and less common disorders, such as ADHD and schizophrenia [2, 10]. The latter set of variants may be better detected with measures of global psychopathology in older children, when thought disorders such as schizophrenia and bipolar disorder occur.

In addition to the genetic correlations with different psychiatric disorders, total psychiatric problems genetically correlated with various psychological and health-related phenotypes, namely intelligence, educational attainment, wellbeing, smoking, body fat and number of children in adulthood. This observation suggests that children genetically predisposed to higher or lower total psychiatric problems are also at risk for or protected against various outcomes related to general development and health. How this correlation arises cannot be inferred from our data, as both vertical and horizontal pleiotropy is plausible. Genetic factors may impact psychiatric problems, which may then lead to adverse health and educational outcomes. However, the reverse is also plausible: genetically determined factors could impact non-psychiatric problems, which then give rise to psychiatric problems. Finally, genetic variants could independently increase the vulnerability to develop problems in many different areas of life. The observed genetic correlations are most likely the result of a combination of these three mechanisms and further research is necessary to examine the causal pathways in detail.

A limitation of this study is the heterogeneity in the measures of psychopathology. On the one hand, using different assessment methods is an advantage, since any associations that are detected will likely be more generalizable. On the other hand, it might limit the ability to detect less robustly associated variants. However, our genetic correlation analyses showed highly consistent effects between cohorts utilizing the CBCL and SDQ questionnaires, which contributed the majority of study participants (65%). Considering that these correlations represent agreement across independent cohorts with numerous methodological and population differences, a genetic correlation above 0.8 provides very strong support for jointly analyzing total psychiatric problems scores assessed by either CBCL or SDQ. The total psychiatric problems assessed by the Rutter questionnaire had a lower genetic correlation with the other assessment methods. However, the Rutter sub-group meta-analysis was only based on three cohorts, representing only 20% of the total study population, which makes it difficult to distinguish instrument effects from other cohort-specific characteristics. Overall, the genetic correlation analyses suggest limited heterogeneity between the majority of cohorts. We thus argue that the lack of power to identify more loci probably stemmed from insufficient sample size, measurement error and the relatively low number of children with severe psychiatric problems in the general population.

Another limitation is that the study sample was not sufficient to consider potential sex interactions. The prevalence of child psychiatric problems differs by gender, with school-age boys typically showing more externalizing problems and girls more internalizing problems. Total scores tend to be less different on average, with boys showing only slightly higher scores [64]. Gene-sex interactions tend to be small for psychiatric disorders and require very high sample sizes to be robustly detected [65]. Future studies with higher sample sizes are therefore needed to identify sex-interactions in the context of total psychiatric problems.

Finally, as in any other GWAS study, the extent to which the found associations can be interpreted causally is difficult. Due to linkage disequilibrium it is unclear whether the two top variants have causal influence on psychopathology or are a marker for other causal variants. The same is true for the association of *SBF2* with total psychiatric problems. However, the association of predicted *SBF2* gene products with neuroticism and psychiatric problems, as well as the influence on myelination in an experimental mouse model, suggest a causal role.

In conclusion, this GWAS of total psychiatric problem scores suggests that common genetic variants exist that are associated simultaneously with internalizing, externalizing, attention and other psychiatric problems in childhood. The pleiotropy was not restricted to psychiatric phenotypes, but also included intelligence, educational attainment, wellbeing, smoking, body fat and number of children in adulthood. Interestingly, we did not find shared genetic effects with autism, schizophrenia and bipolar disorder. Two novel loci were genome-wide significant, though, the low sample size and modest imputation quality necessitate replication before firm conclusions can be drawn whether they influence total psychiatric problems. Furthermore, we found evidence that the gene *SBF2*, which was previously known to be associated with neuroticism, is also implicated in general psychopathology in children. Our results merit further investigation for confirmation and exploration of potential causal mechanisms.

## Supporting information

S1 File Cohort-specific methods. (PDF)

S1 Table Cohort-specific genetic methods. (XLSX)

S2 Table Quality control. (XLSX)

S3 Table Genes with genome-wide suggestive (p<3e-4) results. (PDF)

S4 Table Tissue expression analysis (neural tissues). (PDF)

S5 Table Tissue expression analysis (organs). (PDF)

S6 Table Genetic correlations with thought disorders by assessment age. (PDF)

S1 Fig Quality control steps of individual cohort results. (PNG)

S2 Fig Forest plot of results for rs10767094. Note: SNP only available in subset of cohorts, due to insufficient imputation quality in most studies.
(PNG)

S3 Fig Forest plot of results for rs202005905. Note: SNP only available in subset of cohorts, due to insufficient imputation quality in most studies.
(PNG)

S4 Fig Forest plot of results for rs72854494. (PNG)

S5 Fig QQ-plots gene-based analysis. Quantile-quantile plot of observed -log 10 p values vs expected -log 10 p values assuming chance findings in gene based analysis. Diagonal line indicates a p value distribution compatible with chance finding. Upward deviations indicate p values more significant than expected.
(PNG)

S6 Fig Manhattan plot gene-based analysis. Manhattan plot of -log 10 p values vs SNP position for gene based analysis. Genes above the red horizontal line indicate genome-wide significant findings.
(PNG)
