# Supplementary material for: A genome-wide association study of total child psychiatric problems scores
Source: PLoS One. 2022 Aug 22;17(8):e0273116. doi: 10.1371/journal.pone.0273116 (PMC9394806; doi:10.1371/journal.pone.0273116)
Supplement: S1 File — (PDF) [file pone.0273116.s001.pdf]

Supporting information

Cohort-specific methods

1958 British Birth Cohort

The 1958BC[1] is a population based cohort study initially including all children born in England, Scotland or Wales during one week in March 1958. DNA collection was done at age 45 years. Genome-wide data for the 1958BC was obtained through two sub-studies, both using the 1958BC participants as a control population; one study involved 3,000 randomly selected samples as part of the Wellcome Trust Case Control Consortium (WTCCC), and the other involved 2,592 samples and were used as controls for a type 1 diabetes case-control study (T1DGC). The ethics approval for genetic work was granted by the Joint UCL/UCLH Committees on the Ethics of Human Research (08/H0714/40), written consent was obtained from the participants.

http://www.cls.ioe.ac.uk/page.aspx?&sitesectionid=724&sitesectiontitle=National+Child+Development+Study

ALSPAC

Pregnant women resident in Avon, UK with expected dates of delivery 1st April 1991 to 31st December 1992 were invited to take part in the Avon Longitudinal Study of Parents and Children (ALSPAC).[2,3] The initial number of pregnancies enrolled is 14,541 (for these at least one questionnaire has been returned or a “Children in Focus” clinic had been attended by 19/07/99). Of these initial pregnancies, there was a total of 14,676 foetuses, resulting in 14,062 live births and 13,988 children who were alive at 1 year of age. When the oldest children were approximately 7 years of age, an attempt was made to bolster the initial sample with eligible cases who had failed to join the study originally. As a result, when considering variables collected from the age of seven onwards (and potentially abstracted from obstetric notes) there are data available for more than the 14,541 pregnancies mentioned above. The number of new pregnancies not in the initial sample (known as Phase I enrolment) that are currently represented on the built files and reflecting enrolment status at the age of 24 is 913 (456, 262 and 195 recruited during Phases II, III and IV respectively), resulting in an additional 913 children being enrolled. The phases of enrolment are described in more detail in the cohort profile paper and its update (see footnote 4 below). The total sample size for analyses using any data collected after the age of seven is therefore 15,454 pregnancies, resulting in 15,589 foetuses. Of these 14,901 were alive at 1 year of age.

Please note that the study website contains details of all the data that is available through a fully searchable data dictionary and variable search tool (http://www.bristol.ac.uk/alspac/researchers/our-data/).

Ethical approval for the study was obtained from the ALSPAC Ethics and Law Committee and the Local Research Ethics Committees. Informed consent for the use of data collected via questionnaires and clinics was obtained from participants following the

recommendations of the ALSPAC Ethics and Law Committee at the time. Consent for biological samples has been collected in accordance with the Human Tissue Act (2004).

BREATHE

The BREATHE[4] project (European Commission: FP7-ERC-2010-AdG, ID 268479) is a population-based cohort of primary schoolchildren designed to analyze the association between air pollution and behavior, cognitive function and brain morphology (Sunyer et al. 2015). Thirty-six of the 416 schools in Barcelona were selected based on modeled levels of traffic-related nitrogen dioxide. Thirty-eight schools were located in Barcelona and one school was in an adjacent municipality, Sant Cugat del Vallés. All families of children without special needs who were enrolled in 2nd, 3rd, and 4th grades at the selected schools were invited to participate in the study (2012). A total of 2897 children aged 7 to 11 years accepted the invitation and participated in the project. Genotype data were available for 1667 children of European ethnic origin. All parents or legal guardians gave written informed consent, and the study was approved by the IMIM-Parc de Salut Mar Research Ethics Committee (No. 2010/41221/I), Barcelona, Spain; and the FP7-ERC-2010-AdG Ethics Review Committee (268479-22022011).

http://www.creal.cat/projectebreathe/descripcion.html

CADD

CADD[5] is a longitudinal study of adolescent substance use and associated comorbid conditions in clinical and community cases. Genotyping was done on 1901 subjects, of which 358 had parental CBCL data, and met inclusion criteria. The University of Colorado Human Research Committee approved the study. Participants and their guardians provided informed consent before the start of the study.

https://www.colorado.edu/ibg/human-research-studies/center-antisocial-drug-dependence

CATSS

The Child and Adolescent Twin Study in Sweden (CATSS)[6] is an ongoing longitudinal twin study targeting all twins born in Sweden since July 1, 1992. Subjects are protected by the informed consent process, in which they are informed of what is being collected and repeatedly given the option to withdraw their consent and discontinue their participation. The CATSS-9/12 study has ethical approval from the Karolinska Institute Ethical Review Board: Dnr 03-672 and 2010/507-31/1, CATSS-9 – clinical 2010/1099-31/3 CATSS-15 Dnr: 2009/1599-32/5, CATSS-15/DOGSS Dnr: 03-672 and 2010/1356/31/1, and CATSS-18 Dnr: 2010/1410/31/1.

https://ki.se/meb/catss

COPSAC

The Copenhagen Prospective Studies on Asthma in Childhood 2010 (COPSAC2010)[7] is a clinical birth cohort from Denmark. The $COPSAC_{2010}$ cohort comprises of 700 children

born to unselected mothers from Denmark and has been described previously. The Ethics Committee for Copenhagen and the Danish Data Protection Agency approved this study.

www.copsac.com

FinnTwin12

FinnTwin12[8] is a longitudinal twin study launched in 1994 to investigate the developmental epidemiology of health-related behaviors. From 1994 to 1998, all Finnish families with twins born in 1983–1987 were identified from Finland's Population Register Centre and enrolled into a two-stage sampling design. The first stage included questionnaire assessments of all twins and parents at baseline (87% participation rate, 2724 families) conducted during the year in which the consecutive twin cohorts reached age 11, with follow-up of all twins at ages of 14 and 17½ years, and as young adults (mean age 22). Data collection procedures were approved by the Ethics Committee of Helsinki University Hospital District, Helsinki, Finland and the Institutional Review Board of Indiana University, Bloomington, USA.

www.twinstudy.helsinki.fi

GenR

Generation R[9] is a population-based birth cohort aiming to identify early environmental and genetic determinants of development and health. All parents gave informed consent for their children's participation. The Generation R Study is conducted in accordance with the World Medical Association Declaration of Helsinki and study protocols have been approved by the Medical Ethics Committee of the Erasmus Medical Center, Rotterdam.

www.generationr.nl

Gini-Lisa

The influence of Life-style factors on the development of the Immune System and Allergies in East and West Germany PLUS the influence of traffic emissions and genetics (LISAplus) Study is a population based birth cohort study.[10,11] A total of 3094 healthy, full-term neonates were recruited between 1997 and 1999 in Munich, Leipzig, Wesel and Bad Honnef. The participants were not preselected based on family history of allergic diseases. A total of 5991 mothers and their newborns were recruited into the German Infant study on the influence of Nutrition Intervention PLUS environmental and genetic influences on allergy development (GINIplus) between September 1995 and June 1998 in Munich and Wesel. Infants with at least one allergic parent and/or sibling were allocated to the interventional study arm investigating the effect of different hydrolysed formulas for allergy prevention in the first year of life. All children without a family history of allergic diseases and children whose parents did not give consent for the intervention were allocated to the non-interventional arm. DNA was collected at the age 6 and 10 years and 1511 children from the Munich study center from both studies were genotyped. For both studies, approval by the local Ethics Committees (Ethikkommission der Bayrischen Landeärztekammer, Ethikkommission an der Medizinischen Fakultät der Universität Leipzig, Ärztekammer Nordrhein) and written consent from participant's families were obtained.

https://www.helmholtz-muenchen.de/epi/research/research-groups/allergy-epidemiology/projects/lisa/index.html

https://www.helmholtz-muenchen.de/epi/research/research-groups/allergy-epidemiology/projects/giniplus/index.html

Glaku

The adolescents came from an urban community-based cohort comprising 1049 infants born between March and November 1998 in Helsinki, Finland.[12] Ethics Committees of the City of Helsinki Health Department and Children's Hospital in Helsinki University Central Hospital approved the study protocol. Each child and her/ his parent(s) provided their written informed consent at both follow-ups.

INMA

The INMA—INfancia y Medio Ambiente—(Environment and Childhood) Project[13] is a network of birth cohorts in Spain that aim to study the role of environmental pollutants in air, water and diet during pregnancy and early childhood in relation to child growth and development (http://www.proyectoinma.org/) (Guxens et al. 2012). The study has been approved by Ethical Committee of each participating centre and written consent was obtained from participating parents. Data for this study comes from INMA Sabadell and Valencia subcohorts.

http://www.proyectoinma.org/

MUSP

The Mater Misericordiae Mothers' Hospital-University of Queensland Study of Pregnancy (MUSP)[14] is population-based birth cohort. The first data collection obtained information on 8,556 women. The offspring's development was repeatedly assessed at ages six months, five years, fourteen years and twenty-one years. Focus of the study was the trajectory and determinants of obesity, metabolic syndrome and diabetes, and mental health. The ethics panels of the University of Queensland, Emory University, and the University of California, Los Angeles approved the study and informed consent was obtained by the participants.

https://social-science.uq.edu.au/mater-university-queensland-study-pregnancy

NFBC1986

NFBC1986[15] is a population-based birth cohort aiming to identify early environmental and genetic determinants of development and health. All parents gave informed consent for their children's participation. NFBC1986 is conducted in accordance with the World Medical Association Declaration of Helsinki and study protocols have been approved by the Ethics Committee of Northern Ostrobotnia Hospital District, Finland

www.oulu.fi/nfbc

NTR

NTR[16] is a population-based twin cohort of twins registered shortly after birth by their parents. The Central Ethics Committee on Research Involving Human Subjects of the VU University Medical Center, Amsterdam, and an Institutional Review Board (IRB-2991, NTR 03-180) approved the study. Participants provided Informed consent.

http://www.tweelingenregister.org/

RAINE

The Western Australian Pregnancy Cohort (RAINE) Study[17] is a longitudinal study of 2900 pregnant women and their offspring consecutively recruited from maternity units between 1989 and 1991(2). The inclusion criteria were (i) English language skills sufficient to understand the study demands, (ii) an expectation to deliver at King Edward Memorial Hospital (KEMH), and (iii) an intention to remain in Western Australia to enable future follow-up of their child. Ninety percent of eligible women agreed to participate in the study. From the original cohort, 2868 children have been followed over two decades. The study was conducted with appropriate institutional ethics approval (Human Ethics Committee at KEMH, Princess Margaret Hospital for Children in Perth), and written informed consent was obtained from mothers at all follow-ups and participants at the year 17 follow-up. The RAINE sample is representative of the larger Australian population (88% Caucasian). DNA samples have been collected using standardized procedures at 14 or 16 years of age. Only those children with both biological parents of White European origin were included in the current analyses.

www.rainestudy.org.au

TCHAD

TCHAD[18] is a longitudinal study of all 1480 twin pairs born in Sweden between May 1985 and December 1986 followed with four waves of measurements from childhood (age 8–9), throughout early (age 13–14) and late adolescence (age 16–17), into emerging adulthood (age 19–20). The TCHAD (Dnr 94-277, 98-486, 02-271, 05-628, 12-2107) study has been approved by the Ethics Committee at Karolinska Institutet.

https://ki.se/en/research/swedish-twin-registry-for-researchers

TEDS

TEDS[19] is a multivariate longitudinal study that recruited more than 11,000 twin pairs born in England and Wales in 1994, 1995, and 1996. The TEDS sample is representative of the UK population compared with the data obtained by the Office of National Statistics. The project received approval from the King’s College London Institute of Psychiatry ethics committee, and parental consent was obtained before data collection.

https://www.teds.ac.uk/

TRAILS

TRAILS[20] (TRacking Adolescents' Individual Lives Survey) is a prospective cohort study of Dutch adolescents with bi- or triennial measurements from age 11 to at least age 25 and consists of a general population and a clinical cohort (for a cohort profile see Huisman et al., 2008). In the population cohort, four assessment waves have been completed to date, which ran from March 2001 to July 2002 (T1), September 2003 to December 2004 (T2), September 2005 to August 2007 (T3), and October 2008 to September 2010 (T4). Data for the present study were collected during the third assessment wave. TRAILS is conducted in accordance with the World Medical Association Declaration of Helsinki and the protocol was approved by the Central Committee on Research Involving Human Subjects (CCMO), The Hague, the Netherlands. All participating adolescents and their parents gave written informed consent.

www.trails.nl

YFS

The Young Finns Study[21,22] is an on-going longitudinal population-based cohort study that includes 3 596 healthy Finnish children and adolescents from six birth cohorts (aged 3, 6, 9, 12, 15, and 18 years at the study baseline in 1980). The study was approved by the ethical committee of the Varsinais-Suomi's hospital district's federation of municipalities. For the current study, we selected a subsample of 1 352 participants who had parent-reported psychiatric problems data and genetic data available.

http://youngfinnsstudy.utu.fi/

References

1. Power C, Elliott J. Cohort profile: 1958 British birth cohort (National Child Development Study). *Int J Epidemiol*. 2006;35(1):34-41. doi:10.1093/ije/dyi183
2. Fraser A, Macdonald-Wallis C, Tilling K, et al. Cohort Profile: The Avon Longitudinal Study of Parents and Children: ALSPAC mothers cohort. *Int J Epidemiol*. 2013;42(1):97-110.
3. Boyd A, Golding J, Macleod J, et al. Cohort profile: The 'Children of the 90s'-The index offspring of the avon longitudinal study of parents and children. *Int J Epidemiol*. 2013;42(1):111-127. doi:10.1093/ije/dys064
4. Sunyer J, Esnaola M, Alvarez-Pedrerol M, et al. Association between Traffic-Related Air Pollution in Schools and Cognitive Development in Primary School Children: A Prospective Cohort Study. *PLoS Med*. 2015;12(3):1-24. doi:10.7307/ptt.v30i2.2989
5. Derringer J, Corley RP, Haberstick BC, et al. Genome-Wide Association Study of Behavioral Disinhibition in a Selected Adolescent Sample. *Behav Genet*. 2015;45(4):375-381. doi:10.1007/s10519-015-9705-y
6. Anckarsäter H, Lundström S, Kollberg L, et al. The child and adolescent twin study in Sweden (CATSS). *Twin Res Hum Genet*. 2011;14(6):495-508.
7. Bisgaard H, Vissing NH, Carson CG, et al. Deep phenotyping of the unselected COPSAC2010 birth cohort study. *Clin Exp Allergy*. 2013;43(12):1384-1394. doi:10.1111/cea.12213
8. Rose RJ, Dick DM, Viken RJ, Pulkkinen L, Kaprio J. Drinking or abstaining at age 14? A genetic epidemiological study. *Alcohol Clin Exp Res*. 2001;25(11):1594-1604. doi:10.1111/j.1530-0277.2001.tb02166.x
9. Kooijman MN, Kruithof CJ, van Duijn CM, et al. The Generation R Study: design and cohort update 2017. *Eur J Epidemiol*. 2016;31(12):1243-1264. doi:10.1007/s10654-016-0224-9
10. Berg A V., Krämer U, Link E, et al. Impact of early feeding on childhood eczema: Development after nutritional intervention compared with the natural course - The GINIplus study up to the age of 6 years. *Clin Exp Allergy*. 2010;40(4):627-636. doi:10.1111/j.1365-2222.2009.03444.x
11. Heinrich J, Bolte G, Hölscher B, et al. Allergens and endotoxin on mothers' mattresses and total immunoglobulin E in cord blood of neonates. *Eur Respir J*. 2002;20(3):617-623. doi:10.1183/09031936.02.02322001
12. Strandberg TE, Järvenpää AL, Vanhanen H, McKeigue PM. Birth outcome in relation to licorice consumption during pregnancy. *Am J Epidemiol*. 2001;153(11):1085-1088. doi:10.1093/aje/153.11.1085

13. Guxens M, Ballester F, Espada M, et al. Cohort profile: The INMA-INfancia y Medio Ambiente-(environment and childhood) project. *Int J Epidemiol*. 2012;41(4):930-940. doi:10.1093/ije/dyr054

14. Najman JM, Alati R, Bor W, et al. Cohort profile update: The mater-university of queensland study of pregnancy (MUSP). *Int J Epidemiol*. 2015;44(1):78-78f. doi:10.1093/ije/dyu234

15. Jarvelin M-R, Elliott P, Kleinschmidt I, et al. Ecological and individual predictors of birthweight in a northern Finland birth cohort 1986. *Paediatr Perinat Epidemiol*. 1997;11:298-312.

16. Van Beijsterveldt CEM, Groen-Blokhuis M, Hottenga JJ, et al. The young Netherlands twin register (YNTR): Longitudinal twin and family studies in over 70,000 children. *Twin Res Hum Genet*. 2013;16(1):252-267. doi:10.1017/thg.2012.118

17. McKnight CM, Newnham JP, Stanley FJ, et al. Birth of a cohort — The first 20 years of the raine study. *Med J Aust*. 2012;197(11):608-610. doi:10.5694/mja12.10698

18. Lichtenstein P, Tuvblad C, Larsson H, Carlström E. The Swedish twin study of child and adolescent development: the TCHAD-study. *Twin Res Hum Genet*. 2007;10(1):67-73.

19. Haworth CMA, Davis OSP, Plomin R. Twins early development study (TEDS): A genetically sensitive investigation of cognitive and behavioral development from childhood to young adulthood. *Twin Res Hum Genet*. 2013;16(1):117-125. doi:10.1017/thg.2012.91

20. Huisman M, Oldehinkel AJ, de Winter A, et al. Cohort profile: The Dutch TRacking Adolescents Individual Lives Survey; TRAILS. *Int J Epidemiol*. 2008;37(6):1227-1235. doi:10.1093/ije/dym273

21. Juonala M, Viikari JSA, Raitakari OT. Main findings from the prospective Cardiovascular Risk in Young Finns Study. *Curr Opin Lipidol*. 2013;24(1):57-64. doi:10.1097/MOL.0b013e32835a7ed4

22. Raitakari OT, Juonala M, Rönnemaa T, et al. Cohort profile: The cardiovascular risk in young Finns study. *Int J Epidemiol*. 2008;37(6):1220-1226.
