# Supplementary material for: A genome-wide association study of total child psychiatric problems scores
Source: PLoS One. 2022 Aug 22;17(8):e0273116. doi: 10.1371/journal.pone.0273116 (PMC9394806; doi:10.1371/journal.pone.0273116)
Supplement: S3 Table — (PDF) [file pone.0273116.s004.pdf]

Table S3: Genes with genome-wide suggestive (p<3e-4) results

Gene	Chr	BP Start	BP Stop	n_{snps}	n	p
IFT46	11	118415243	118443685	43	35725	3E-05
MAN1A1	6	119498374	119670926	495	36917	8E-05
ARCN1	11	118443105	118473748	49	34777	9E-05
TMEM25	11	118401756	118417995	35	36873	1E-04
SPATA7	14	88851268	88936694	58	35794	1E-04
C14orf180	14	105046021	105056852	24	31043	1E-04
PTPN21	14	88932122	89021077	130	36435	1E-04
OR2G6	1	248684916	248685964	1	25907	1E-04
TBCA	5	76986991	77164604	454	36311	1E-04
FBXO46	19	46213887	46234162	17	33100	2E-04
TMEM56	1	95582894	95663163	184	35599	2E-04
ARTN	1	44398992	44402913	5	35680	2E-04
IPO13	1	44412611	44433694	21	36631	2E-04
MUC5B	11	1244296	1283406	102	31472	2E-04
TRPS1	8	116420724	116821899	428	36001	3E-04
SCGB1C1	11	193080	194573	4	19491	3E-04
LDHAL6A	11	18477371	18501147	52	32796	3E-04

Chr Chromosome
BP Basepair
Start Basepair Position of gene start
Stop Basepair Position of gene end
n_{snps} Number of SNPs within the gene
n Number of participants
p p-value
