# Supplementary material for: A genome-wide association study of total child psychiatric problems scores
Source: PLoS One. 2022 Aug 22;17(8):e0273116. doi: 10.1371/journal.pone.0273116 (PMC9394806; doi:10.1371/journal.pone.0273116)
Supplement: S4 Table — (PDF) [file pone.0273116.s005.pdf]

Table S4: Tissue expression analysis (neural tissues)

Brain Region	β	$β_{SD}$	SE	p	q
Caudate basal ganglia	0.013	0.022	0.007	0.04	0.12
Putamen basal ganglia	0.013	0.022	0.007	0.04	0.12
Anterior cingulate cortex	0.012	0.021	0.007	0.04	0.12
Amygdala	0.012	0.021	0.007	0.05	0.12
Hypothalamus	0.012	0.021	0.008	0.05	0.12
Nucleus accumbens	0.011	0.019	0.007	0.06	0.12
Hippocampus	0.011	0.019	0.007	0.07	0.12
Frontal Cortex BA9	0.009	0.017	0.006	0.07	0.12
Cortex	0.008	0.015	0.007	0.11	0.16
Substantia nigra	0.008	0.015	0.008	0.15	0.19
Cerebellar Hemisphere	0.005	0.010	0.006	0.18	0.21
Cerebellum	0.004	0.008	0.006	0.25	0.27
Spinal cord cervical c-1	0.000	0.000	0.008	0.51	0.51

β Beta
$β_{SD}$ Beta Standard Deviation
SE Standard Error
p P-value
q False Discovery Adjusted P-values
