# Supplementary material for: A genome-wide association study of total child psychiatric problems scores
Source: PLoS One. 2022 Aug 22;17(8):e0273116. doi: 10.1371/journal.pone.0273116 (PMC9394806; doi:10.1371/journal.pone.0273116)
Supplement: S5 Table — (PDF) [file pone.0273116.s006.pdf]

Table S5: Tissue expression analysis (organs)

Tissue	β	$β_{SD}$	SE	p	q
Brain	0.02	0.03	0.01	0.06	0.81
Liver	0.01	0.01	0.01	0.08	0.81
Breast	0.02	0.03	0.01	0.13	0.81
Pituitary	0.01	0.03	0.02	0.18	0.81
Adipose tissue	0.00	0.01	0.01	0.23	0.81
Nerve	0.01	0.01	0.01	0.26	0.81
Kidney	0.01	0.01	0.01	0.28	0.81
Skin	0.01	0.01	0.01	0.28	0.81
Muscle	0.01	0.01	0.01	0.29	0.81
Vagina	0.01	0.01	0.01	0.30	0.81
Bladder	0.00	0.01	0.01	0.32	0.81
Colon	0.01	0.01	0.01	0.32	0.81
Stomach	0.00	0.00	0.01	0.38	0.87
Testis	0.00	0.00	0.02	0.48	0.87
Blood	0.00	0.00	0.01	0.48	0.87
Pancreas	0.00	0.00	0.01	0.48	0.87
Salivary Gland	0.00	0.00	0.01	0.50	0.87
Cervix Uteri	0.00	0.00	0.01	0.59	0.94
Small intestine	0.00	-0.01	0.02	0.62	0.94
Blood vessel	0.00	0.00	0.01	0.63	0.94
Prostate	-0.01	-0.01	0.02	0.66	0.94
Heart	-0.01	-0.01	0.01	0.71	0.94
Uterus	-0.01	-0.01	0.01	0.72	0.94
Esophagus	-0.01	-0.02	0.01	0.78	0.94
Spleen	-0.01	-0.02	0.01	0.82	0.94
Adrenal gland	-0.01	-0.02	0.01	0.86	0.94
Fallopian tube	-0.01	-0.02	0.01	0.88	0.94
Thyroid	-0.02	-0.03	0.01	0.91	0.94
Lung	-0.02	-0.03	0.01	0.91	0.94
Ovary	-0.02	-0.03	0.01	0.96	0.96

β Beta
$β_{SD}$ Beta Standard Deviation
SE Standard Error
p P-value
q False Discovery Rate Adjusted P-values
