# Supplementary material for: A genome-wide association study of total child psychiatric problems scores
Source: PLoS One. 2022 Aug 22;17(8):e0273116. doi: 10.1371/journal.pone.0273116 (PMC9394806; doi:10.1371/journal.pone.0273116)
Supplement: S6 Table — (PDF) [file pone.0273116.s007.pdf]

Table S6: Genetic correlations with thought disorders by assessment age

Total Problems Age Group	Correlated trait	PMID	r_G	SE	p
<12 years	Schizophrenia	25056061	0.06	0.04	0.07
≥12 years	Schizophrenia	25056061	0.11	0.06	0.08
<12 years	Bipolar disorder	21926972	-0.09	0.07	0.17
≥12 years	Bipolar disorder	21926972	-0.02	0.10	0.85

PMID PubMed ID, $\mathbf{r_G}$ Genetic Correlation, **SE** Standard Error, **p** P-value,
