# Supplementary figures and images for: A genome-wide association study of total child psychiatric problems scores
Source: PLoS One. 2022 Aug 22;17(8):e0273116. doi: 10.1371/journal.pone.0273116 (PMC9394806; doi:10.1371/journal.pone.0273116)

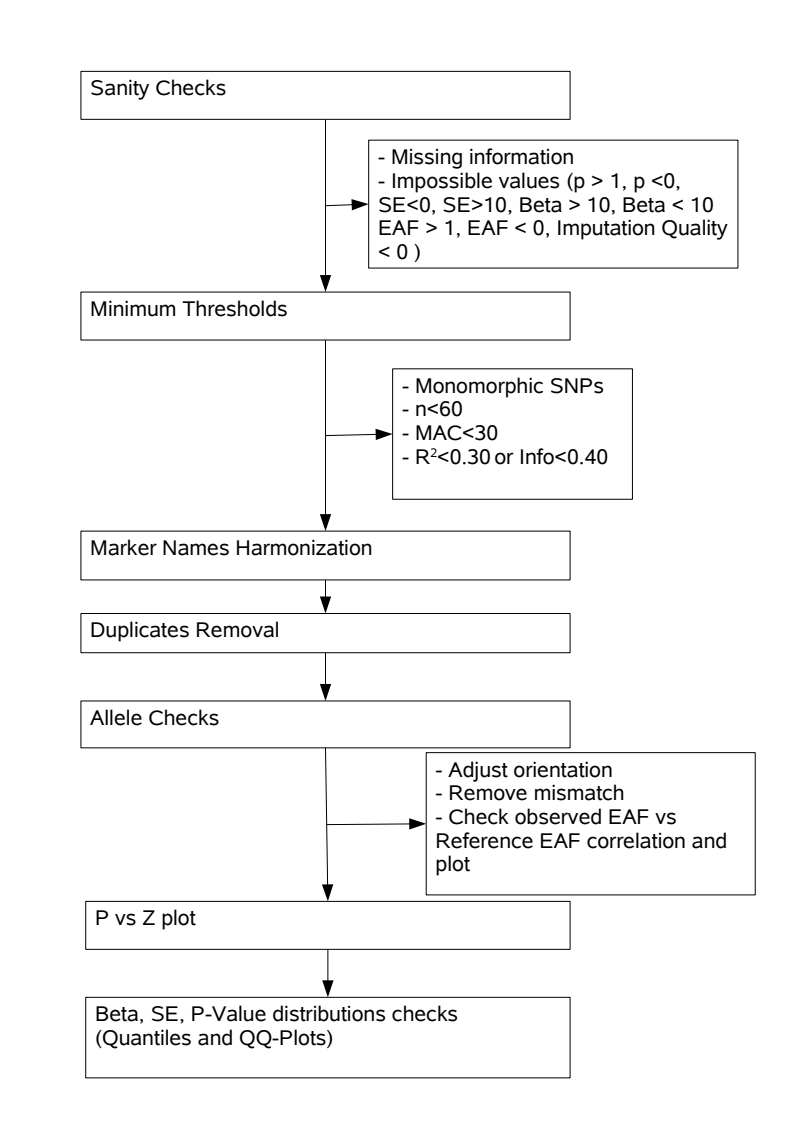

Supplement: S1 Fig — (PNG) [file pone.0273116.s008.png]

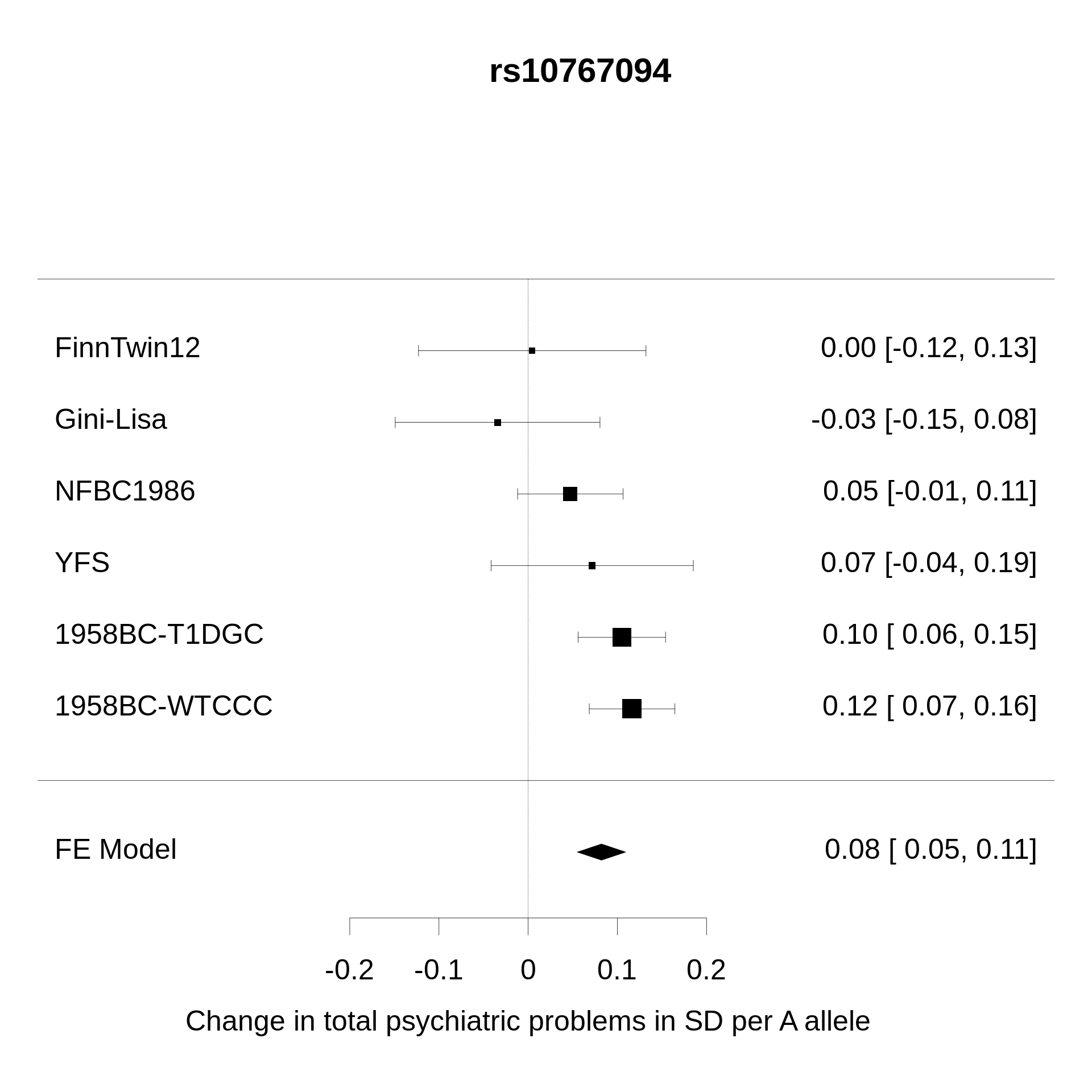

Supplement: S2 Fig — Note: SNP only available in subset of cohorts, due to insufficient imputation quality in most studies. (PNG) [file pone.0273116.s009.png]

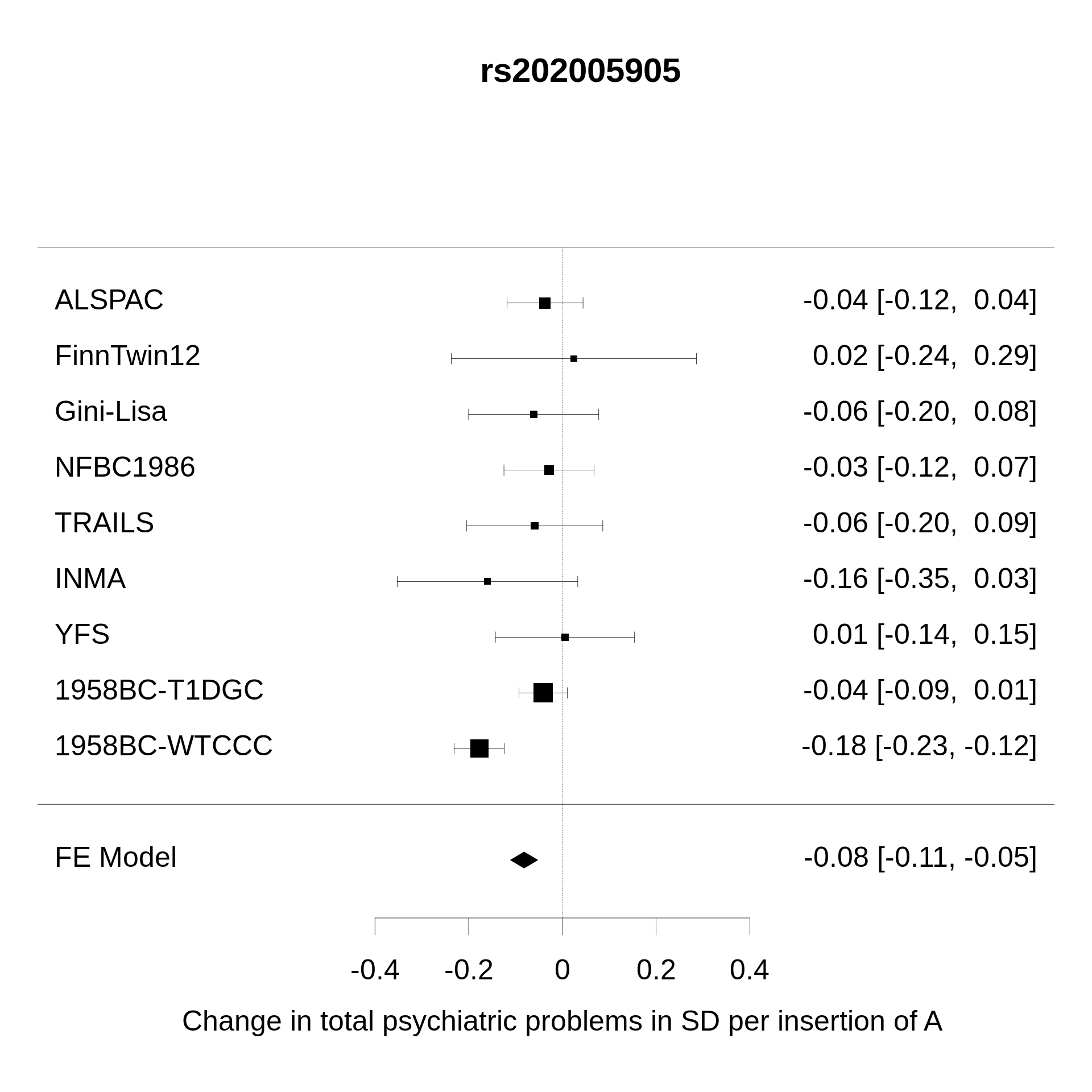

Supplement: S3 Fig — Note: SNP only available in subset of cohorts, due to insufficient imputation quality in most studies. (PNG) [file pone.0273116.s010.png]

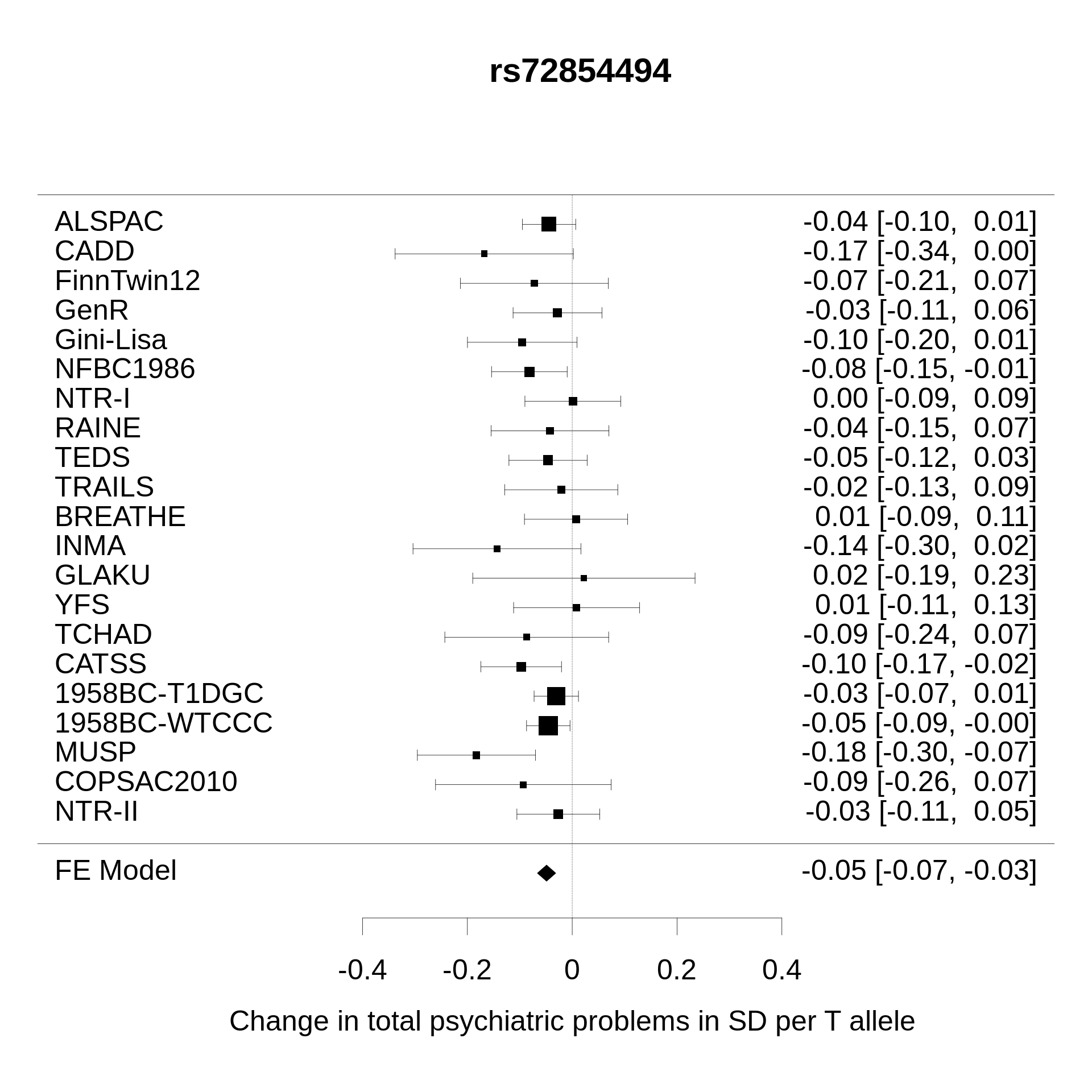

Supplement: S4 Fig — (PNG) [file pone.0273116.s011.png]

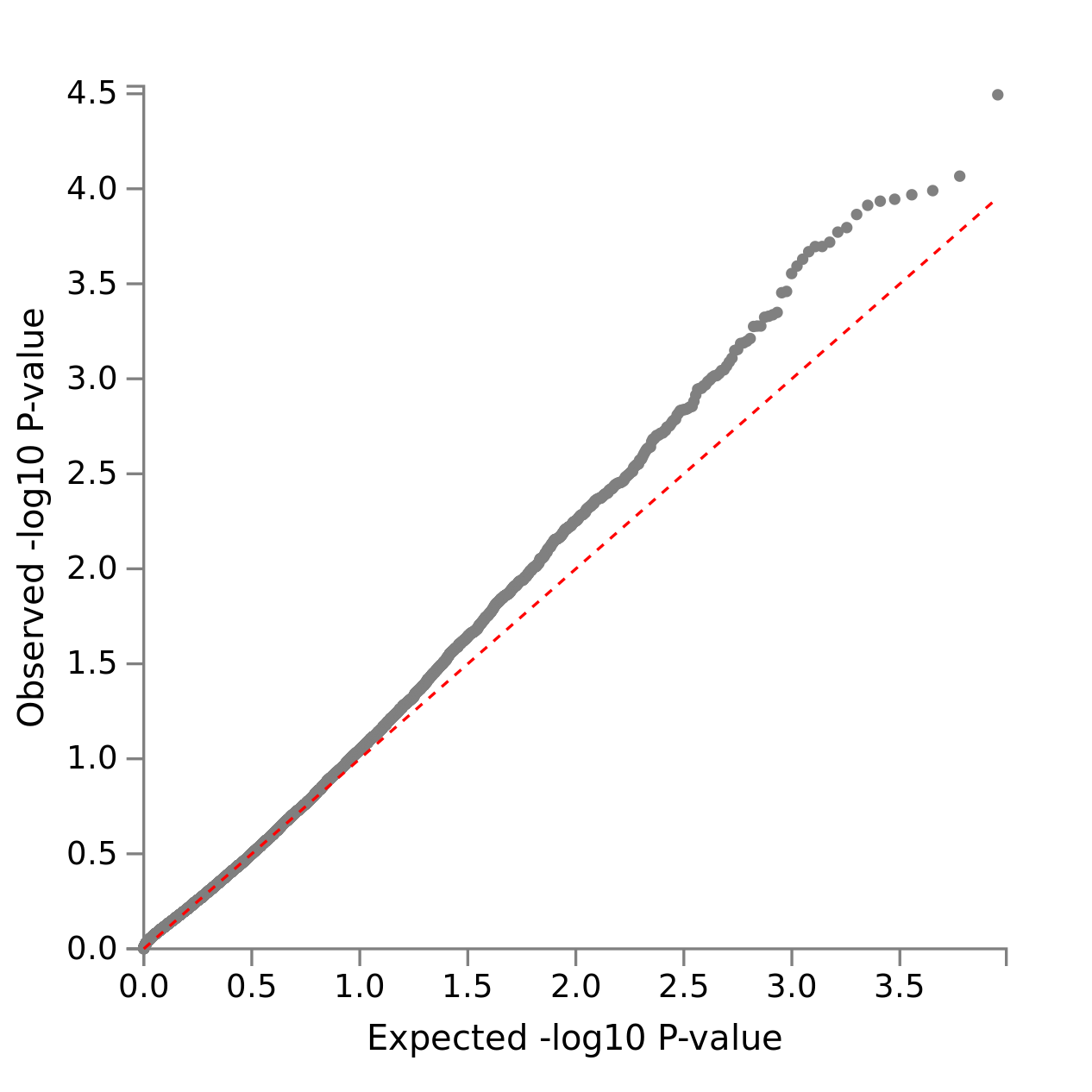

Supplement: S5 Fig — Quantile-quantile plot of observed -log 10 p values vs expected -log 10 p values assuming chance findings in gene based analysis. Diagonal line indicates a p value distribution compatible with chance finding. Upward deviations indicate p values more significant than expected. (PNG) [file pone.0273116.s012.png]

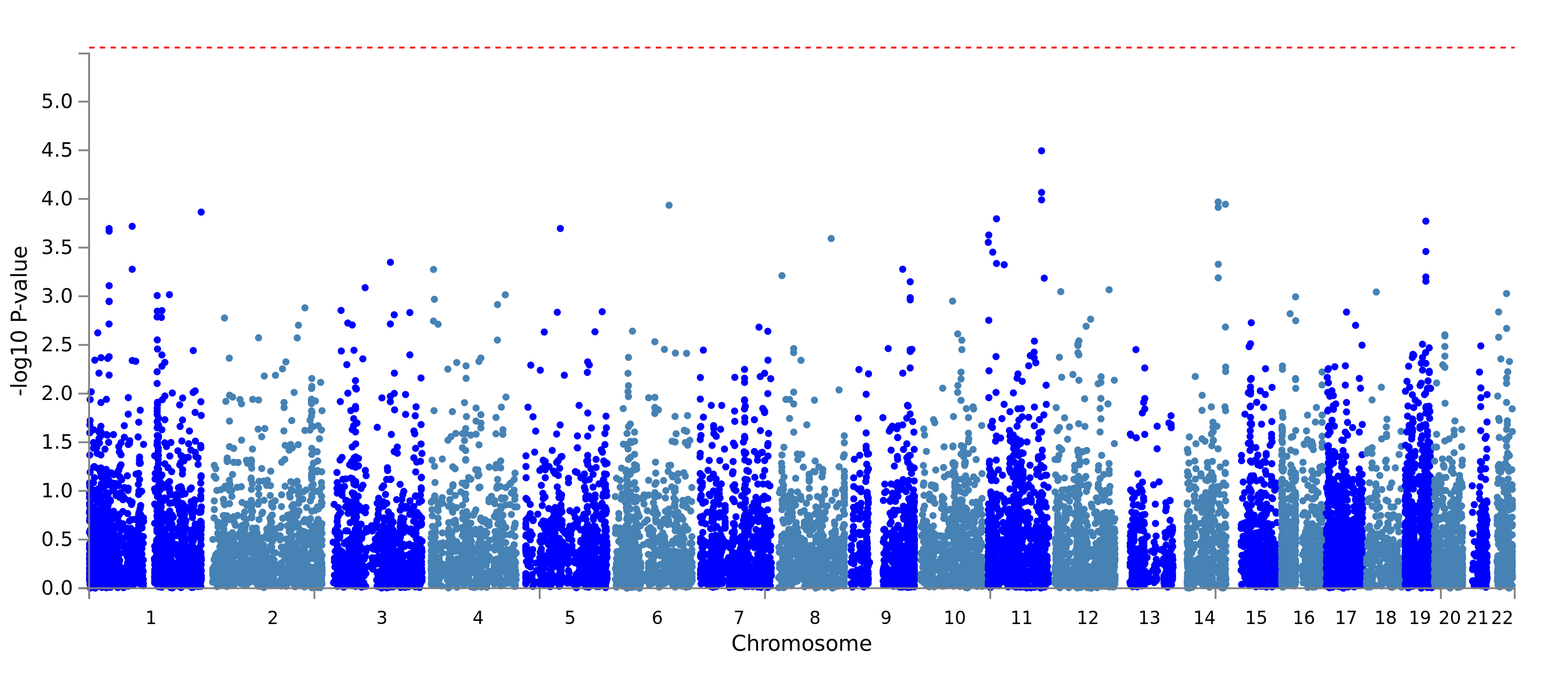

Supplement: S6 Fig — Manhattan plot of -log 10 p values vs SNP position for gene based analysis. Genes above the red horizontal line indicate genome-wide significant findings. (PNG) [file pone.0273116.s013.png]
